# *In vitro* P38MAPK inhibition in aged astrocytes decreases reactive astrocytes, inflammation and increases nutritive capacity after oxygen-glucose deprivation

**DOI:** 10.18632/aging.202651

**Published:** 2021-02-09

**Authors:** Miren Revuelta, Amaia Elicegui, Till Scheuer, Stefanie Endesfelder, Christoph Bührer, Leire Moreno-Cugnon, Ander Matheu, Thomas Schmitz

**Affiliations:** 1 Department for Neonatology, Charité University Medical Center, Berlin 13353, Germany; 2 Cellular Oncology Group, Biodonostia Health Research Institute, Paseo Doctor Begiristain, San Sebastian 20014, Spain; 3 Neurovascular Research Laboratory, Vall d’Hebron Institute of Research, Barcelona 08035, Spain; 4 IKERBASQUE, Basque Foundation for Science, Bilbao 48013, Spain; 5 CIBERfes, Madrid 28029, Spain

**Keywords:** astrocytes, OGD, ageing, p38MAPK

## Abstract

Proper astroglial functioning is essential for the development and survival of neurons and oligodendroglia under physiologic and pathological circumstances. Indeed, malfunctioning of astrocytes represents an important factor contributing to brain injury. However, the molecular pathways of this astroglial dysfunction are poorly defined. In this work we show that aging itself can drastically perturb astrocyte viability with an increase of inflammation, cell death and astrogliosis. Moreover, we demonstrate that oxygen glucose deprivation (OGD) has a higher impact on nutritive loss in aged astrocytes compared to young ones, whereas aged astrocytes have a higher activity of the anti-oxidant systems. P38MAPK signaling has been identified to be upregulated in neurons, astrocytes and microglia after ischemic stroke. By using a pharmacological p38α specific inhibitor (PH-797804), we show that p38MAPK pathway has an important role in aged astrocytes for inflammatory and oxidative stress responses with the subsequent cell death that occurs after OGD.

## INTRODUCTION

Ischemic stroke, the most common type of stroke, represents the second main cause of death worldwide leading to 5.7 million of deaths per year in adulthood [1, 2]. Between all types of stroke, brain ischemic stroke represent 87% of all cases that cause neurological deficits such as motor impairment and inability to read or even aphasia [3].

During the last decades, ischemic stroke experimental models have been performed in young animals, but aging itself plays a critical role in the response of the brain to stroke [4]. Indeed, brain aging is accompanied by many structural and physiological alterations that usually involve cognitive decline [5]. Although most of the molecular mechanisms of brain injury are similar in neonatal and aged animal models [6, 7], certain characteristics are different, e.g., cell apoptosis occurs a week after the injury in the immature brain while this period takes place only for s few hours after the damage in the mature brain [8]. For *in vitro* studies, the commonly used model to mimic cerebral ischemia is the oxygen glucose deprivation model (OGD) [9, 10].

Although neurons are primarily susceptible to injury, impairment of supporting glial cells such as astrocytes may contribute to secondary injury in neurons [11]. Proper astroglial functioning is essential for the development and survival of neurons and oligodendroglia under pathological circumstances [12]. Supportive properties of astrocytes are reflected in production of growth factors such as PDGF and IGF [13, 14], clearance of radicals via superoxide dismutase activity [15], anti-oxidant defense with glutathione synthesis [16], removal of glutamate from the synaptic cleft [17], among others. Hence, malfunctioning of astrocytes represents an important factor for recovery and repair of the brain after injury [18, 19]. The importance of age for differences in glial cellular responses after brain injury were also documented in microglia [20].

p38 mitogen-activated protein kinase (p38MAPK) pathway is a signaling pathway that can be activated in neurodegenerative diseases [21, 22]. p38MAPK controls key processes of mammalian cell homeostasis such as self-renewal, differentiation, proliferation and death [23]. Activation of p38MAPK signaling has been identified in neurons, astrocytes and microglia after ischemic stroke [24]. Moreover, p38MAPK inhibitors seem to be effective reducing infarct volume after stroke [21, 25, 26], but the cell type being involved in p38MAPK dependent injury in the brain after stroke remains to be elucidated, as well as the effect of p38MAPK in aged cells after stroke.

Therefore, our aim was to investigate the effect of OGD in young and aged primary rat astrocyte cultures and to analyze the expression and effect of p38MAPK in these cultures. For this, we cultured young and aged astrocytes with PH-797804, MAPK14 (*p38α)* inhibitor, the most abundant isoform of p38MAPK in the brain, to define changes in supporting and protective properties of astrocytes that can be critical for survival of brain cells.

## MATERIALS AND METHODS

### Animals

All animal experiments were performed in accordance to German and Spanish animal welfare law with the permission of the Animal Welfare Committee of Berlin (LAGeSo T-0124/08) and the permission of Biodonostia Institute Animal Care Committee. Wistar rats (FEM, Charité) were housed in specific pathogen-free barrier areas of the Hospital Charité Institute in Berlin while the C57B/6 (Jackson Laboratory) mice were housed in the Biodonostia Institute. Mice were maintained under a 12-hour light/12 hour dark cycle at 22° C with controlled humidity and with food and water provided ad libitum and handled in compliance with the animal research regulations specified in the European Communities Directive [2010/63/EU].

### Astrocyte primary cultures

Primary astrocytes cell culture was prepared from neonatal Wistar rats during the first postnatal day of life. After dissection and careful removal of the meninges, both hemispheres were dissociated mechanically [13]. Cells were resuspended in Dulbecco’s Modified Eagle Medium (DMEM, Invitrogen, USA) supplemented with 20% fetal calf serum, 1% penicillin/streptavidin and subsequently seeded in T75 flasks coated with poly-L-lysine (200 μg/ml, Sigma-Aldrich) and grown in a humidified incubator maintained at 37° C under >90% humidity and 5% CO_2_. Medium was changed every 2-3 days. After 7-10 days, the cultures were shaken overnight to minimize oligodendroglia and microglia contamination. For purification, the remaining astrocyte monolayers were trypsinized and replated. Cells were seeded in 12-well plates containing approximately 15,000 cells for immunocytochemistry and ATP analysis; in 6-well plates containing approximately 500,000 cells for quantitative RT-PCR and 10^6^ cells/well for western blot analysis.

### Oxygen-glucose deprivation (OGD)

The culture medium was replaced with deoxygenated and glucose-free DMEM. Then cells were transferred to a humidified anaerobic chamber filled with a gas mixture of 5% CO_2_ and 95% N_2_ at 37° C. Throughout the OGD period of 4 hours, an atmosphere of <0.5% oxygen was maintained. After the OGD period, the media was quickly replaced with glucose-containing (1g/L) DMEM. Some samples were collected and others were reintroduced into normoxic conditions for 20 hours of reperfusion/recovery. Control cells in DMEM containing 4.5g/L glucose were always kept in a normoxic incubator.

OGD was performed 24h after cells were seeded (1DIV) or 4 weeks after (30DIV) to simulate an *in vitro* model of aging astrocytes [20]. The medium of the aged cells was changed every 2-3 days. For assays with P38MAPK inhibitor, astrocytes were treated with 2 μM PH-797804 (Selleckchem) added to the medium when seeded and included in every medium change. Control group were astrocytes with and without treatment in the different stages but without OGD.

### ATP detection

ATP concentration was detected with the use of the Luminescent ATP Detection Assay Kit (Abcam, Cambridge, UK) according to the manufactures guide lines. Briefly, cultured astrocytes were incubated with a detergent on a shaker for 5 minutes. After total cell lyses, substrate for luminescence detection was added and incubated for 5 minutes. Luminescence was analyzed by IVIS Lumina *in vivo* Imaging System (PerkinElmer).

### Tissue immunofluorescence

After mice perfusion, coronal serial sections of 50 μm were collected via SM2010 R Sliding Microtome (Leica) from young (2 months-old) and aged (2 years old) C57BL/6 mice and selected brain sections were blocked with 10% donkey serum and 0.1% Triton X-100 in phosphate-buffered saline and incubated with anti-P-p38MAPK (1:200; rabbit, Cell Signalling) and anti-GFAP conjugated with Alexa Fluor 488 (1:500; mouse, Sigma) overnight at 4° C. Nuclei were stained with DAPI staining (Sigma). Images were acquired using a Leica confocal microscope.

### Cell immunofluorescence

After removal of the medium, cells seeded on coverslips were fixed with 4% paraformaldehyde for 15 min for immunocytochemical procedure. The samples were pre-incubated with a blocking solution (10% goat serum and 0.1% Triton X-100 in phosphate-buffered saline) for 30 min and then incubated with a mouse monoclonal anti-GFAP (1:400, Sigma, USA) for 2 hrs at room temperature, followed by incubation with rabbit anti cleaved caspase-3 (Asp175) (1:400, cell signaling) at 4° C overnight. After washes, cells were incubated with the respective secondary antibodies. Finally, after three washings, the sections were mounted with Vectashield HardSet Mounting Medium with DAPI (Sigma). Images were acquired using a Leica confocal microscope.

### Quantitative RT-PCR

RNA extraction was performed with acidic phenol/chloroform (peq-GOLDRNApure, PEQLAB Biotechnologie, Erlangen, Germany) following the manufacturer protocol, and 2 μg of RNA were reverse transcribed. Primers were used for insulin-like growth factors (*Igf*), neuronal growth factor (*Ngf*), glial fibrillary acidic protein (*Gfap*), superoxide dismutase 2 (*Sod2*), glutamine synthetase (*Gs*), glutamate aspartate transporter (Slc1a3), catalytic subunit of glutamate cysteine ligase (*Gclc*), to analyse the cDNA and *Hprt* was used as housekeeping gene (see Table 1). The expressions of target genes were analyzed with the StepOnePlusTM qPCR System (Applied Biosystems, Life Technologies, Carlsbad, CA) according to the 2−ΔΔ*C*T method [27].

**Table 1:** Primer used in qPCR.

**Gene**	**Forward primer**	**Reverse primer**	**GenBank ID**
**Cntf**	AACCTTGACTCAGTGGATGGTGTA	AAGCCTGGAGGTTCTCTTGGA	NM_013166.1
**Gclc**	GGAGGACAACATGAGGAAACG	GCTCTGGCAGTGTGAATCCA	NM_012815.2
**Gfap**	TCTGGACCAGCTTACTACCAACAG	TGGTTTCATCTTGGAGCTTCTG	NM_017009.2
**Igf-1**	CGGACCAGAGACCCTTTGC	GCCTGTGGGCTTGTTGAAGT	NM_001082479.1
**Ngf**	ACCCAAGCTCACCTCAGTGTCT	GACATTACGCTATGCACCTCAGAGT	NM_001277055.1
**Slc1a3**	CCCTGCCCATCACTTTCAAG	GCGGTCCCATCCATGTTAAT	NM_001289942.1
**Sod2**	GACCTACGTGAACAATCTGAACGT	AGGCTGAAGAGCAACCTGAGTT	NM_017051.2
**Hprt**	GGAAAGAACGTCTTGATTGTTGAA	CCAACACTTCGAGAGGTCCTTTT	NM_012583.2

### Western blot analysis

Immunoblots were performed following standard procedures. Equal amounts of protein (20 μg) were separated on 15% SDS polyacrylamide gels and blotted onto nitrocellulose membranes (BioRad). Primary antibodies were P-p38MAPK (1:200; Cell Signalling), total GFAP (1:100, Santa Cruz), TNFα (1:200, PromoCell), cleaved caspase 3 (1:1000, Cell Signaling) and α-ACTININ (1:2000, Sigma), followed by appropriate secondary antibodies conjugated with horseradish peroxidase (DAKO). Detection was performed by chemiluminescence using ECL (Amersham).

### Statistical analysis

All data are presented as mean ± standard error of the mean (SEM). After the assessment of normality with Kolmogorov Smirnov test (KS), t student test was performed to compare the differences between the control and the OGD group. Group differences were studied by one-way ANOVA with Bonferroni-Dunn correction. Two-sided P<0.05 was considered statistically significant. The statistical analysis of data was performed using GraphPad prism 5 software version 5.01 (GraphPad Software, Inc. CA, USA).

## RESULTS

### Aged astrocytes show increased GFAP reactivity, inflammation, and cell death as well as lower nutritive and anti-oxidative capacity

**Figure 1:**
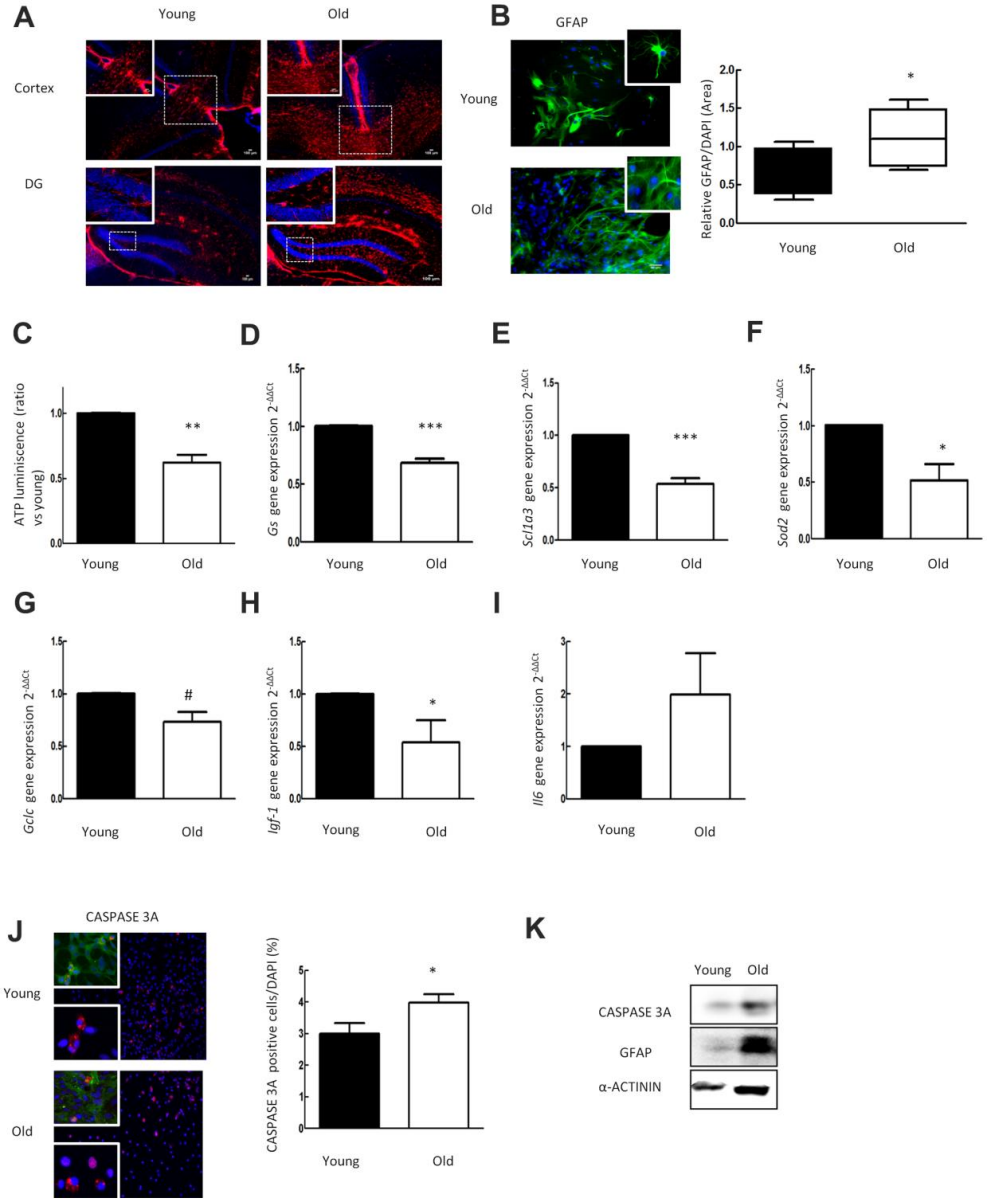
**Aged astrocytes have increased reactivity, inflammation and cell death as well as loss of nutritive and anti-oxidative capacity.** (**A**) Representative immunofluorescence for GFAP (red) in cortex and DG of young (2 month-old) and aged (over 24 month-old) C57BL/6J mice (n=2). (**B**) Representative immunofluorescence and the quantification for GFAP positive cells in 1DIV (young) and 30DIV (old) primary astrocytes cell culture derived from neonatal Wistar (n=6). (**C**) ATP luminescence levels of young and old primary astrocytes cultures (ratio compared to the young group) (n=4). (**D**–**I**) Expression of *gs, slc1a3, sod2, gclc, igf-1* and *il-6* in young and old primary astrocytes cultures (n=6). (**J**) Representative immunofluorescence of CASPASE 3A and co-staining of CASPASE 3A (red) with GFAP (green) together with DAPI (blue). Quantification for CASPASE 3A positive cells and in 1DIV (young) and 30DIV (old) primary astrocytes cell culture derived from P1 Wistar rat pups (n=6). (**K**) Protein expression of CASPASE 3A and GFAP in 1DIV (young) and 30DIV (old) primary astrocytes cell culture. Results are expressed as the mean ± SEM. Asterisks denote the significance levels when compared to the control group (***p<0.001, **p<0.01 and *p<0.05 versus controls, t-test).

We determined the GFAP reactivity in the cortex and dentate gyrus (DG) of the hippocampus in young (2 month-old) and aged (≥ 2 year-old) *C57BL/6* mice. Immunofluorescence showed an increased expression of GFAP with aging (Figure 1A). To verify the age dependent increase of GFAP in astrocytes, we cultured rat neonatal astrocytes and simulated an *in vitro* model of aging (30DIV). Increased GFAP expression *in vivo* was confirmed in primary astrocytes *in vitro*. Aged astrocytes showed increased GFAP expression (0.6 ± 0.13 vs 1.3 ± 0.15 ratio vs DAPI) compared to younger cells (1DIV) (Figure 1B).

Mitochondria generate the majority most of cellular ATP supply, which is essential for energy consuming cell processes [28]. To investigate the impact of aging on cellular energy supply we measured the ATP concentration in 30DIV (old) astrocytes and 1DIV (young). As a result, ATP luminescence was reduced in aged astrocytes to almost half of the level found in young cells (P<0.01) (Figure 1C).

Regulation of glutamate homeostasis is one of the major functions of astrocytes in the brain. The enzyme glutamine synthetase (GS) is needed to transform glutamate intracellularly into glutamine, which is stored in vesicles [29]. In order to determine the impact of aging on glutamate homeostasis, we analyzed gene expression of *Gs* and of *Slc1a3* (EAAT1 or GLAST) representing a glutamate transporter molecule highly abundant in these cells [30]. qPCR analysis revealed that expression of *Gs* and *Slc1a3* is drastically reduced in aged astrocytes (Figure 1D, 1E). These results demonstrate that aging alters the expression of genes relevant for glutamate homeostasis in astrocytes.

The astrocytes anti-oxidant defense system is regulated via the Nrf2-pathway [31], which orchestrates the expression of its target genes including glutathione synthetase [32]. To define the impact of aging on anti-oxidant gene expression in astrocytes, we determined levels of *Sod2* and of catalytic subunit of glutamate cysteine ligase (*Gclc*) gene expression. The expression levels of *Sod2* and *Gclc* were significantly decreased in aged cultures indicating impairment of the anti-oxidant system in old astrocytes (Figure 1F, 1G).

To further characterize the mechanism of cell alterations caused by aging, we determined the expression of various growth factors, inflammatory response related genes, and performed caspase-3a (CASP3A) immunofluorescence staining for apoptosis. Aged astrocytes displayed a reduction of *igf* and an increase of *il6* (Figure 1H, 1I) gene expression. The numbers of CASP3A positive astrocytes in aged cultures were increased as compared to young cultures (2.9 ± 0.5 % vs. 3.9 ± 0.3 %, number of CASP3A^+^ cells expressed as a percentage of all DAPI cells (p<0.05) (Figure 1J). Western blot analysis corroborated the results of elevated GFAP and CASP3A expression in old cells (Figure 1K).

### Loss of function in response to OGD is more pronounced in aged than in young astrocytes: role of cellular ATP levels, nutritive properties, and anti-oxidative capacity

**Figure 2:**
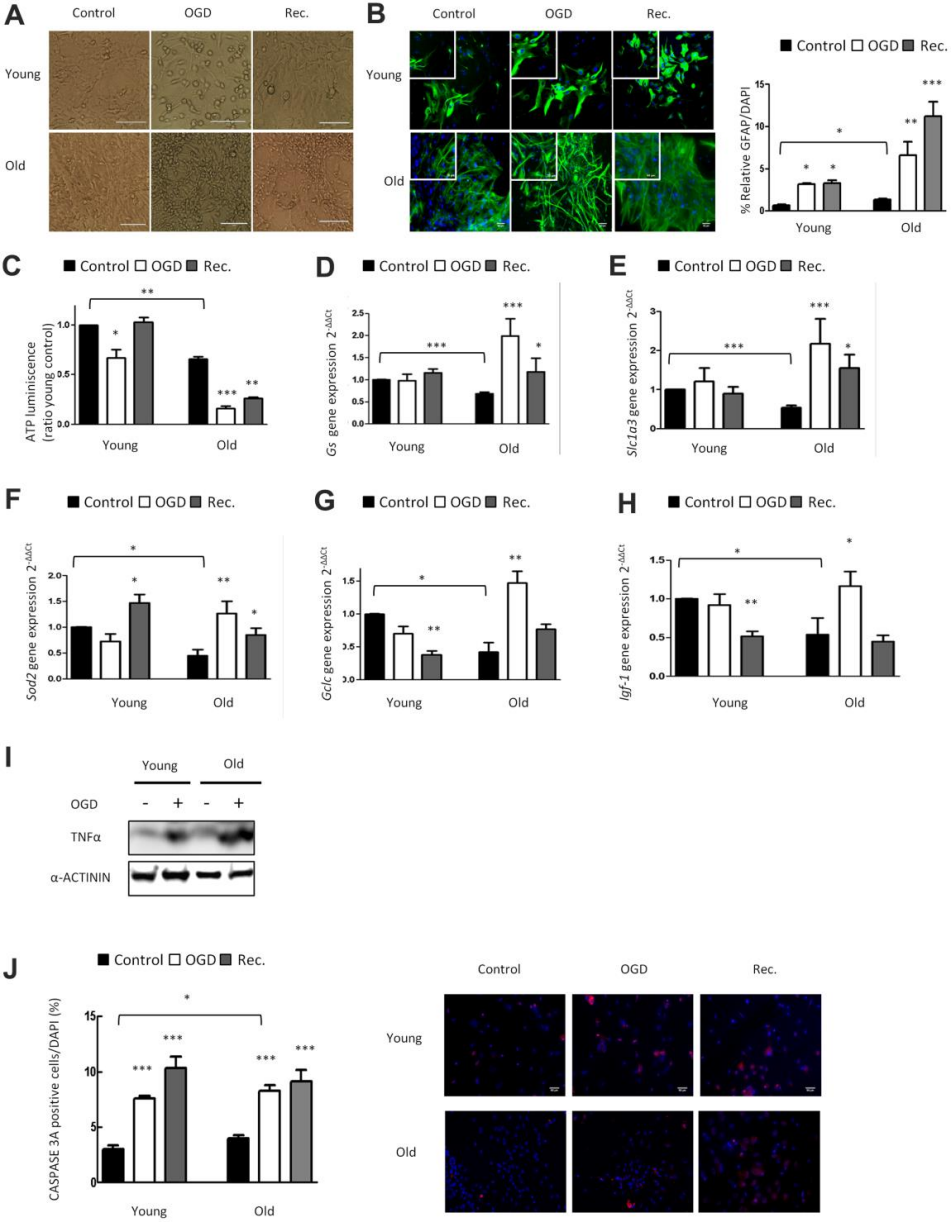
**OGD has a higher impact in nutritive loss in aged compared to young astrocytes, whereas aged astrocytes have better anti-oxidant systems.** (**A**) Representative optical microphotograph of young and old primary astrocytes derived from neonatal Wistar rats after 4h of OGD and after 20h of recovery compared to the control group. (**B**) Representative immunofluorescence and quantification of GFAP positive cells (green) in 1DIV (young) and 30DIV (old) primary astrocyte cell cultures after 4h of OGD and after 20h of recovery compared to the age matched control groups without OGD (n=6). (**C**) ATP luminescence levels in young and old primary astrocyte cultures after 4h of OGD and after 20h of recovery compared to control groups (n=4). (**D**–**H**) Expression of *gs, slc1a3, sod2, gclc* and *igf-1* in young and old astrocytes after 4h of OGD and after 20h of recovery compared to controls (n=6). (**I**) Protein expression of TNFα in young and old cultured astrocytes after 4h of OGD compared to controls. (**J**) Representative immunofluorescence and the quantification for CASPASE 3A positive cells in 1DIV (young) and 30DIV (old) astrocytes cell culture derived from neonatal Wistar after 4h of OGD and after 20h of recovery compared to the control group (n=6). Results are expressed as the mean ± SEM. Asterisks denote the significance levels when compared to the control group (***p<0.001, **p<0.01 and *p<0.05 versus controls, t-test).

To investigate the impact of OGD in young and aged astrocytes *in vitro*, we performed 4h of OGD (Figure 2A), and determined GFAP immunoreactivity in young and aged astrocytes. Immunofluorescence showed a significant OGD-induced increase of GFAP expression in both groups after OGD, which was even more pronounced in aged astrocytes (Figure 2B). The staining of GFAP was elevated exclusively in aged astrocytes after reperfusion/recovery.

We also determined the effect of OGD on cellular energy supply in young and aged astrocytes by ATP assay (Figure 2C). Reduction of ATP dependent energy after OGD in young astrocytes was restored back to control levels after 20 hours of reperfusion/recovery. Notably, in aged astrocytes, the OGD-induced decline of energy supply was still largely reduced after the phase of recovery (Figure 2C). This highlights that OGD hits aged astrocytic energy supply in a persistent way, and that the capacity of recovery and cellular repair is strongly impaired in old as compared to young astrocytes.

We also characterized the effect of OGD on glutamate uptake, anti-oxidative capacity, growth factor regulation, inflammatory response and cell death in young and aged astrocytes. Aging caused a significant upregulation of Gs, Slc1a3, Sod2, Gclc immediately after OGD indicating astroglial activation and induction of the anti-oxidant defense system compared to young astrocytes (Figure 2D–2G). OGD increased Igf-1 expression only in aged astrocytes but its expression was downregulated after reperfusion/recovery in both groups (Figure 2H). Finally, there was a significant upregulation of TNFα and CASPASE3A after OGD in young and aged astrocytes (Figure 2I, 2J).

### p38MAPK activity increases in aged astrocytes and its expression is reduced with PH-797804

**Figure 3:**
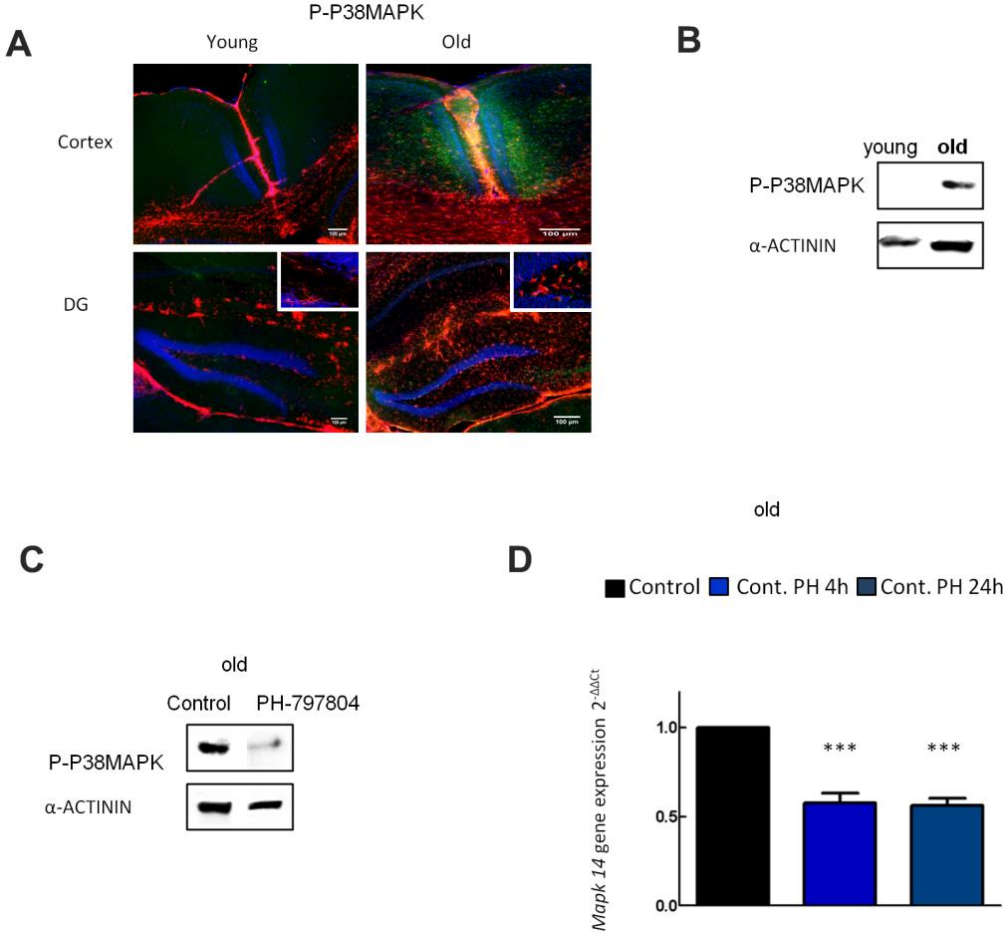
**p38MAPK activity increases in aged astrocytes and its expression is reduced with PH-797804.** (**A**) Representative immunofluorescence and quantification for phosphorylated p38MAPK (P-p38MAPK) (green) together with DAPI (blue) and GFAP (red) in the cortex and in the DG of young (2 month-old) and aged (over 24 month-old) C57BL/6J mice (n=2). (**B**) Immunoblot of P-p38MAPK in 1DIV (young) and 30DIV (old) primary astrocyte cultures derived from Wistar rat brains. (**C**) Immunoblot of P-p38MAPK in old astrocyte cultures with and without PH-797804 treatment. (**D**) MAPK14 gene expression in old astrocyte cultures treated with PH-797804 at different time points in comparison to the control groups (without treatment) (n=6). Results are expressed as the mean ± SEM. Asterisks denote the significance levels when compared to the control group (***p<0.001, **p<0.01 and *p<0.05 versus controls, t-test).

p38MAPK activity (P-p38MAPK) and its isoforms are increased in different cell types in aged mouse brain [33]. Indeed, p38MAPK is one of the most important kinases in inflammatory signaling and its activation has been identified in many neurodegenerative diseases [21]. We first studied the expression of p38MAPK in the cortex and DG of hippocampus in young (2 month-old) and aged (≥ 2 year-old) C57BL/6J mice. Immunofluorescence showed that P-p38MAPK expression was increased in over 2 year-old animals (Figure 3A), supporting previous studies in the DG [33]. Next, we measured P-p38MAPK in cultured young and aged astrocytes and found that P-p38MAPK expression was strongly increased in aged compared to young astrocytes (Figure 3B). Finally, the addition of PH-797804 to aged astrocyte cultures lowered levels of P-P38MAPK and *Mapk14* (p38alpha) gene expression by 50% after OGD and after reperfusion in comparison to control cells (Figure 3C, 3D).

### Pharmacological inhibition of p38α in aged astrocytes prevents astroglial reactivity, inflammatory response and anti-oxidant defense system activation after OGD

**Figure 4:**
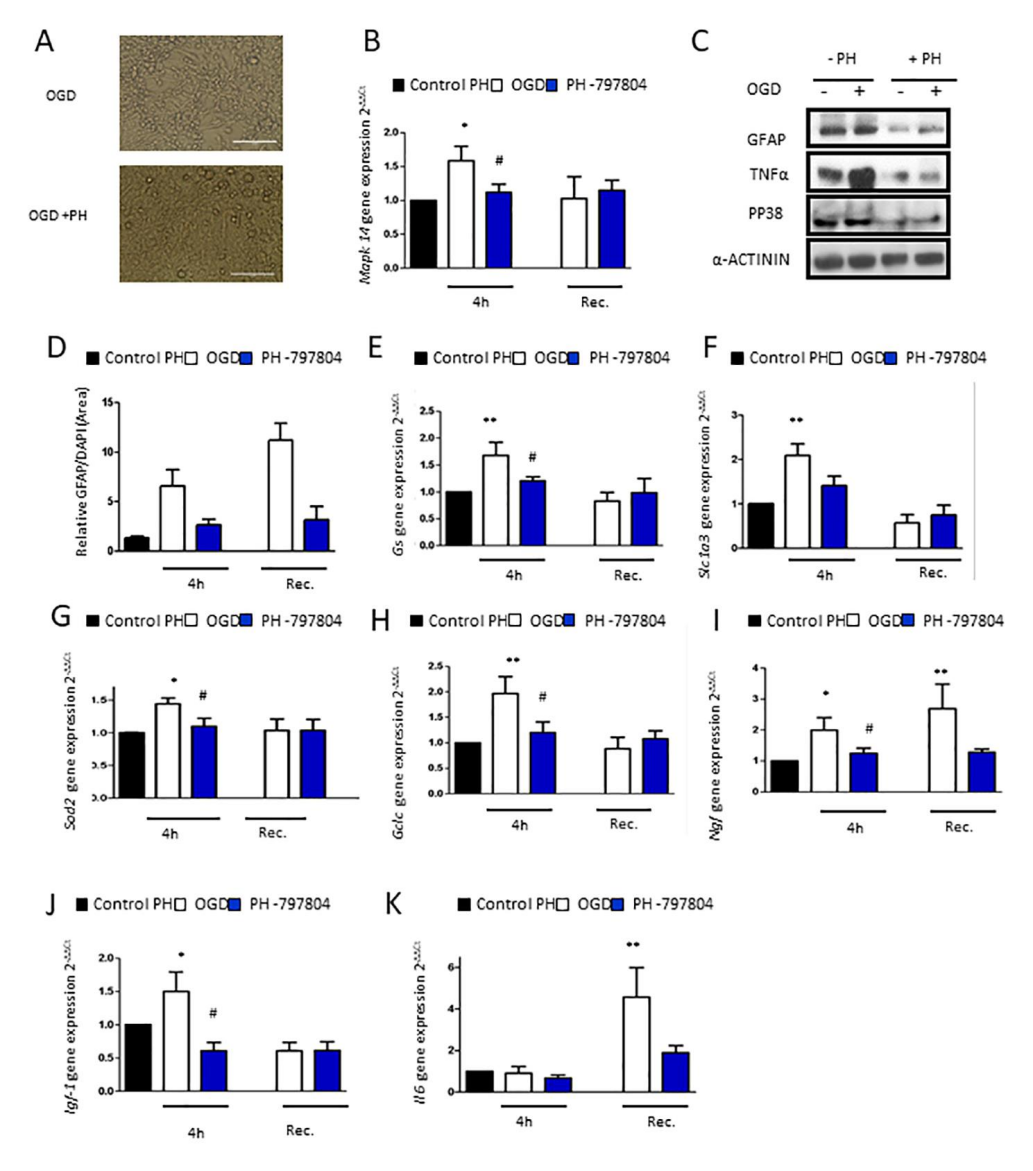
**Pharmacological inhibition of p38α in aged astrocytes prevents from astrocytes reactivity, inflammatory response and anti-oxidant defense system activation after OGD.** (**A**) Representative optical microphotograph of old primary astrocytes derived from neonatal Wistar after 4h of OGD and treated with PH-797804. (**B**) MAPK14 gene expression in old astrocyte cultures after 4h of OGD (white), after 4h of OGD with PH-797804 treatment (blue column) in comparison to controls treated with PH-797804 (black) (n=6). (**C**) Immunoblot of GFAP, TNFα and P-p38MAPK in old primary astrocytes cell culture derived from neonatal Wistar in normoxia and after 4h of OGD and with or without the treatment, PH-797804. (**D**) Representative quantification of GFAP positive cells in old astrocyte cultures derived after 4h of OGD, after 4h of OGD with PH-797804 treatment, and in control cultures treated with PH-797804 (n=6). (**E**–**K**) Expression of *gs, slc1a3, sod2, gclc, ngf, igf-1* and *il-6* in old astrocyte after 4h of OGD, after 4h of OGD with PH-797804 treatment, in controls treated with PH-797804 (n=6). Results are expressed as the mean ± SEM. Asterisks denote the significance levels when compared to the control group (***p<0.001, **p<0.01 and *p<0.05 versus controls, t-test).

Finally, we aimed to clarify whether p38α inhibition could have beneficial effects in aged astrocytes after 4h of OGD (Figure 4A). We first detected that *Mapk14* (p38MAPK alpha) expression was increased after OGD in aged astrocyte compared to the control group (Figure 4B). Analysis of TNFα, GFAP and P-p38MAPK protein expression after OGD revealed that there was a reduction of all these proteins in aged astrocytes treated with PH-797804 after OGD (Figure 4C).

We corroborated astroglial inhibition with GFAP immunofluorescence in which astrocytes treated with the p38α inhibitor expressed lower levels of astrogliosis compared to the untreated OGD group directly after the injury and also after recovery (Figure 4D).

To further characterize the effect of pharmacological inhibition of p38α in aged astrocytes after OGD and after recovery, we characterize the effects on glutamate uptake, anti-oxidative capacity and growth factor response. Gene expression of selected genes of those processes, *Gs, Slc1a3, Gclc, Sod, Ngf* and *Igf-1* was reduced in p38α inhibited astrocytes treated with PH-797804 immediately after OGD and also after recovery (Figure 4E–4J). Hence, pharmacological inhibition of p38α attenuates the oxidative challenge in aged astrocytes caused by OGD.

Finally, we analyzed the inflammatory response of astrocytes with PH-797804 treatment after OGD. The increase in aged astrocytes after 20h of recovery was fully abolished after pharmacological p38α inhibition (Figure 4K).

## DISCUSSION

In the central nervous system (CNS), astrocytes are the central cell type to support neuronal survival under pathological circumstances [34]. Indeed, as astrocytes, but not neurons, shift in gene expression patterns with aging, it has been proposed that they may be a better indicator of age in the brain than neurons [35]. Our *in vitro* results showed that aging increases the reactivity and inflammatory response of primary rat astrocytes. Previous reports show that astrocytes show age-dependent inflammatory responses [36] and increased GFAP expression [37, 38], which has been characterized as a hallmark of brain aging [5]. Moreover, our results indicate that the anti-oxidant and cellular energy system of *in vitro* aged astrocytes is decreased compared to the young ones, hence facilitating increased cell death. A proper cellular anti-oxidative capacity is necessary for oxidative stress protection, and a decline in ATP levels may contribute to neurodegeneration as it is related to regulating neuronal activity through synaptic inhibition [39]. The challenge through sublethal energy failure in our *in vitro* experiments may therefore lead or contribute to exhaustion of the anti-oxidant system. Furthermore, we found decreased *Gs* and *Scl1a3* expression in aged astrocytes, hence pointing towards perturbed glutamate homeostasis as a potential trigger of cellular toxicity [29]. As a consequence, it can be assumed that protection of neurons via the anti-oxidant system of astrocytes is impaired by aging [40]. As a nutritive factor, IGF1 is considered to be essential for neuronal survival and development [41]. Moreover, previous studies have found that a lack of IGF1 reduces brain size with a cellular loss in the neuronal population and myelination deficits [42], but little attention has been paid to astrocytes as a source of IGF1, specifically. Consequently, the *Igf1* reduction found in our aged astrocytes may represent an injurious event to brain cells that were previously hit by energy failure. Altogether, the complex changes including inflammatory response, astrogliosis, energy failure, decreased anti-oxidative capacity and elevated caspase-3 activity may contribute to an increased “*in vitro*” aged astrocyte cell death.

As mentioned, aging alone increases astroglial reactivity [43], which is an observation also supported by our results, but this glial response is exaggerated following ischemia-stroke in the brain, hence accelerating glial scar formation [4]. We found that responses of energy failure and inflammation were exaggerated following 4 hours of OGD in our *in vitro* aged astrocytes compared to the young astrocytes, and that these alterations were maintained during the phase of recovery. However, although aged astrocytes present decreased anti-oxidative capacity, after OGD we observed an upregulation of anti-oxidant system defense, growth factor release and glutamate uptake. Cellular defense in the brain involve endogenous protective enzymes such as SOD and glutathione (GSH), which are produced by astrocytes to protect neurons against oxidative stress. In previous studies it has been demonstrated that the cellular anti-oxidant system and GSH release is upregulated in astrocytes as a response to pathological circumstances, possibly as a defense mechanism to prevent cell apoptosis [44]. Hence, the increase of cellular anti-oxidant defense system, *Gs* and *Slc1a3* expression and growth factor expression may represent a cellular attempt of compensation in aged astrocytes in response to astrogliosis, inflammation and CASPASE 3 activation induced by OGD.

Our results show enhanced p38MAPK activity with aging in astrocytes, both in astrocyte cultures *in vitro* and in the cortex and dentate gyrus (hippocampus) *in vivo,* which complement previous results obtained in neurogenic niches (SVZ) *in vivo* and in neurospheres derived from aged mice [45]. Elevated p38MAPK activity has also been identified in neurodegenerative disease and in response to brain injury such as brain stroke [46], and its pharmacological inhibition ameliorated symptoms of neurodegenerative diseases and was protective against ischemia [21, 25]. We show that *Mapk14* expression was increased in aged astrocytes directly after OGD and that pharmacological p38α inhibition in aged astrocytes after OGD restored *Mapk14* expression back to control levels. Moreover, the P38MAPK pathway has been described as inflammatory mediator in the CNS [33]. In our results, the inactivation of p38α in aged astrocyte cultures treated by PH-797804 attenuated astroglial activation and inflammation that occur after OGD. We also found that PH-797804 treatment in aged astrocytes can prevent OGD-induced changes of growth factors *igf* and *ngf*, of the free radical clearance factors *sod2* and *gclc,* and of the glutamate metabolism system *gs* that were increased after OGD. The impaired production of these factors has previously been reported to alter the supportive properties of astrocyte [13, 47].

In summary, our experiments with OGD highlight an impairment of various astroglial functions that are important for the support of neuronal development and for protection of the brain both in young and aged astrocytes cell culture. Inhibition of p38α in aged *in vitro* astrocytes may provide effective protection reducing astrogliosis and inflammation that occurs in aged astrocyte after OGD event.

### Ethics approval

All procedures were approved by the state animal welfare authorities Berlin, Germany (LAGeSo T-0124/08) and followed institutional guidelines.
